# Pampas deer (*Ozotoceros bezoarticus*) courtship and mating behavior

**DOI:** 10.1186/1751-0147-54-60

**Published:** 2012-10-13

**Authors:** Jéssica T Morales-Piñeyrúa, Rodolfo Ungerfeld

**Affiliations:** 1 Departamento de Fisiología, Facultad de Veterinaria, Montevideo, Uruguay

**Keywords:** Cervid, Reproductive behavior, Sexual ethogram, Proceptivity, Ruminant

## Abstract

**Background:**

Pampas deer, *Ozotoceros bezoarticus* (Linnaeus 1758), is a South American grazing deer categorized as "near threatened". However, knowledge about pampas deer behavior including courtship and mating is scarce and incomplete. The aim of this study was to characterize the courtship and mating behavior of the pampas deer (*Ozotoceros bezoarticus*), an endangered species from South America.

**Methods:**

We performed focal observations of 5 males allocated at the Estación de Cría de Fauna Autóctona Cerro Pan de Azúcar, Uruguay, 4 times a day from 5 to 20 minutes each time on a daily basis from February to May. During that period we recorded all courtship and mating behaviors, as well as quantified the frequency of the specific behaviors shown. As mating were rarely observed, we recorded that behavior when it was observed in the context of other studies performed in the same population during the following 2 years.

**Results:**

During the observation period we recorded 928 courtships and 5 mating periods. In addition, we recorded 10 more matings performed during other studies, totaling 15. The duration of each mating calculated from the 15 recordings was 3.9 ± 0.4 s, and the total period of female receptivity (from first to last mating acceptance) was 8.2 ± 1.1 min. Main observed courtship behaviors in males were “chase” and “ostentation”, while the most observed close to mating were “chinning”, “raised head” and “anogenital sniffing”. The most observed behaviors in females during the mating period were “vulva exhibition” and “move away”.

**Conclusion:**

This is the first detailed report in pampas deer mating behavior. Estrus lasted only 8 min accepting only 3 short copulations per estrus. However, female behavior during courtship can be characterized as highly proceptive.

## Background

Most studies on deer sexual behavior report that females progressively modify their behavior as a response to male courtship, and finally stay immobile when the male approaches [1-5]. The first mating indicates the onset of estrus, which generally lasts approximately 24 h [6-8]. In white-tailed, red, and wapiti deer each mating lasts 5 to 15 sec [1,9].

Pampas deer, *Ozotoceros bezoarticus* (Linnaeus 1758), is a South American grazing deer. The IUCN [10] categorized this species as “near threatened” and listed it in Appendix I of the Convention on International Trade in Endangered Species of Wild Fauna and Flora [11]. In Uruguay there are two wild populations, and a third one bred in semi-captivity since 1980 located at the Estación de Cría de Fauna Autóctona (ECFA), Maldonado. This species presents many particularities probably related to its evolution in grassland areas [12]. However the knowledge about its sexual behavior is scarce and incomplete [9,13-15]. The most complete description of courtship behavior of free living pampas deer was published by Verdier [16] in a local monograph but because of practical difficulties in studying animals that could not be identified, that study only describes general behavioral patterns without individual recordings. Matings were not recorded in any of the referenced studies. Ungerfeld *et al*. [12] presented a complete description of courtship behavior but reported only a description of one mating. Therefore, basic sexual patterns concerning the length and number of mounts during heat and behaviors displayed near mating have not been reported before.

Free living pampas deer form groups in which several males may be continuously in contact with a group of females without clear sexual segregation throughout the year [17,18]. In polyandrous species such as the pampas deer, length of estrus is related to breeding strategies. In long estrous period species, in which different males may mate with a single female, post-mating sperm competition should be the main determinant of paternity. On the other hand, short mating acceptance periods decrease the risk of post-mating sperm competition, emphasizing the strategies by which males gain access to estrous females. Considering all this information, the aim of this study was to characterize pampas deer sexual behavior, including the male and female courtship and mating behaviors.

## Methods

The study was conducted at the ECFA, Uruguay (34° 3’ S, 55° 1’ W) with 5 groups of animals housed in 0.5 to 1 ha pens with shade, trees and shrubs. Each group was composed of one adult male, 5 to 9 adult females, and young animals. Pens were near each other (minimum distances = 3 m), allowing visual, auditory, and olfactory contact among animals allocated in different pens. Animals grazed native grasses and received approximately 600 g of a concentrate for dairy cows/animal/day. All animals were identified with numbered tags and were used to human presence. They allowed us to observe them from a distance of 5-6 m.

According to previous observations [12] female breeding season occurs between February and May (late summer-early autumn). Therefore, focal observations [19] of the 5 males with harem females were performed by one observer for periods of 5 to 20 min per group, 4 times daily, all days of those months. Observations were performed twice in the morning (between 8:00 and 11:00 am) and twice in the afternoon (between 4:00 and 7:00 pm). If behavior indicated that mating might occur, observations continued until mating was confirmed or discarded, or until the end of the daylight. As mating were rarely observed, we recorded that behavior when observed in the context of other studies performed in the same population during the following 2 years. The methodology used remained unchanged during this follow-up period. Observations of mating periods continued until receptiveness ended, and the same female was frequently observed during the following hours to be unreceptive to further mating attempts.

Three trained observers accomplished the data (separately) by direct observation or using binoculars avoiding permanently to disturb the animal’s normal behaviors. We recorded on real-time all behaviors performed by each male, or by male-female dyads. The overall observations performed 260 hours in the February through May period.

Male courtship behaviors are those that involve an active sexual display from the male toward a female. The mating period included the behaviors displayed immediately before, during, and after the mating, ending when the female was no longer receptive and the interactions between male and female stopped. We developed a basic sexual ethogram analyzing the real-time data and the videos. Considering the previous descriptions available from other widely studied deer species, and other small ruminants, including red deer [2], mouflon [20] and sheep [21], as well as preliminary information reported in pampas deer [12,16] we performed male courtship and male and female mating ethograms. The frequency in which each behavior was displayed in different contexts (males during courtship and males and females during mating periods) was calculated. We also analyzed the average number of mounts per mating period, and the length of the receptive period and of each mating.

## Results

### General description

We recorded 928 courtship and 5 estrus during the studied period (February to May), and we recorded another 10 estrus periods during the next 2 years. Each mate lasted 3.9 ± 0.4 s (mean ± SEM), and the total period of mating acceptance was 8.2 ± 1.1 min. In those periods females accepted 2.0 ± 0.2 mounts (range 1 to 3). During the recorded period after the end of the receptive period, no female accepted new mating attempts. In the analysis, the data from a juvenile female (7 months old) were excluded as they could not be considered representative (it accepted10 mounts during a single180 min period).

### Courtship behavior

We identified and described eight male courtship behaviors: chivying, low stretch, follow sniffing, chasing, ostentation, flehmen, anogenital sniffing and licking, and guard, all of which were also observed during the mating periods. From these, 6 behaviors reinforce and expand previous descriptions and 2 have not been reported before for the species. We present the description of each behavior in Table 1. High frequencies of chases and ostentation (Table 1), and long-term guards characterized courtship. During this period, the only female responses observed were slow walks or quick races to get away when the male chased her. Anogenital sniffing and licking were rarely observed (Table 1). As the courtship progressed, the activity displayed by the female toward the male increased.

**Table 1:** Description and mean frequencies of the courtship behaviors (mean ± SEM) displayed by 5 pampas deer males (total = 928 records)

**Behaviors**	**Description**	**Recordings**
Chase	The male chased the female by trotting behind her while the female ran, vocalizing several times in high tone.	24.3 ± 1.3
Ostentation	The male remained standing immobile in front of the female, sometimes walking a few steps toward her, with his head and neck outstretched. The chin was held up high, look fixed, stamping alternating his forelegs on the ground firmly, and simultaneously making snort like noises.	15.6 ± 3.4
Guard	The male lay down near the female (1 to 2 m), and may stayed there for several hours	12.8 ± 2.0
Low stretch	The male walked toward the female with his head and neck stretched, the head held in a low position, a few centimeters above the ground, maintaining a distance of less than 2 m (Figure 1), accompanied by frequent vocalizations. At the same time it could perform tongue movements.	12.1 ± 1.7
Flehmen	The male raised its head, the external nares were drew back and with the mouth opened upper lip was curled back.	10.1 ± 1.6
Chivying	The female was more than 5 m far from the male, and the male marched toward the female, with the neck in a normal position. Occasionally the male vocalized.	9.7 ± 1.5
Follow sniffing	The male lowered and extended his head and neck following the female, both walking or chasing, sniffing the ground, with the ears up, maintaining a distance of less than 2 m. The movements were accompanied by acute vocalizations.	8.6 ± 1.7
Anogenital sniffing and licking	The male pushed its face into the female's anogenital region and sniffed and/or licked it.	8.2 ± 1.9

### Mating behaviors

The male sexual ethogram included the following behaviors:

•“Sniffing and licking of the body”: the male’s nose approached any part of the female´s body, either sniffed or licked female’s body while she stood quiet.

•“Chinning”: when the female stayed immobile, the male approached from behind and chinned and throated on the female´s back, performing a light downward pressure.

•“Mount attempt”: the male attempted to mount the female, generally from her side or back, performing extension and retraction movements of the tongue. Penis protrusion may accompany this behavior. No intromission of the penis occurred.

**Figure 1:**
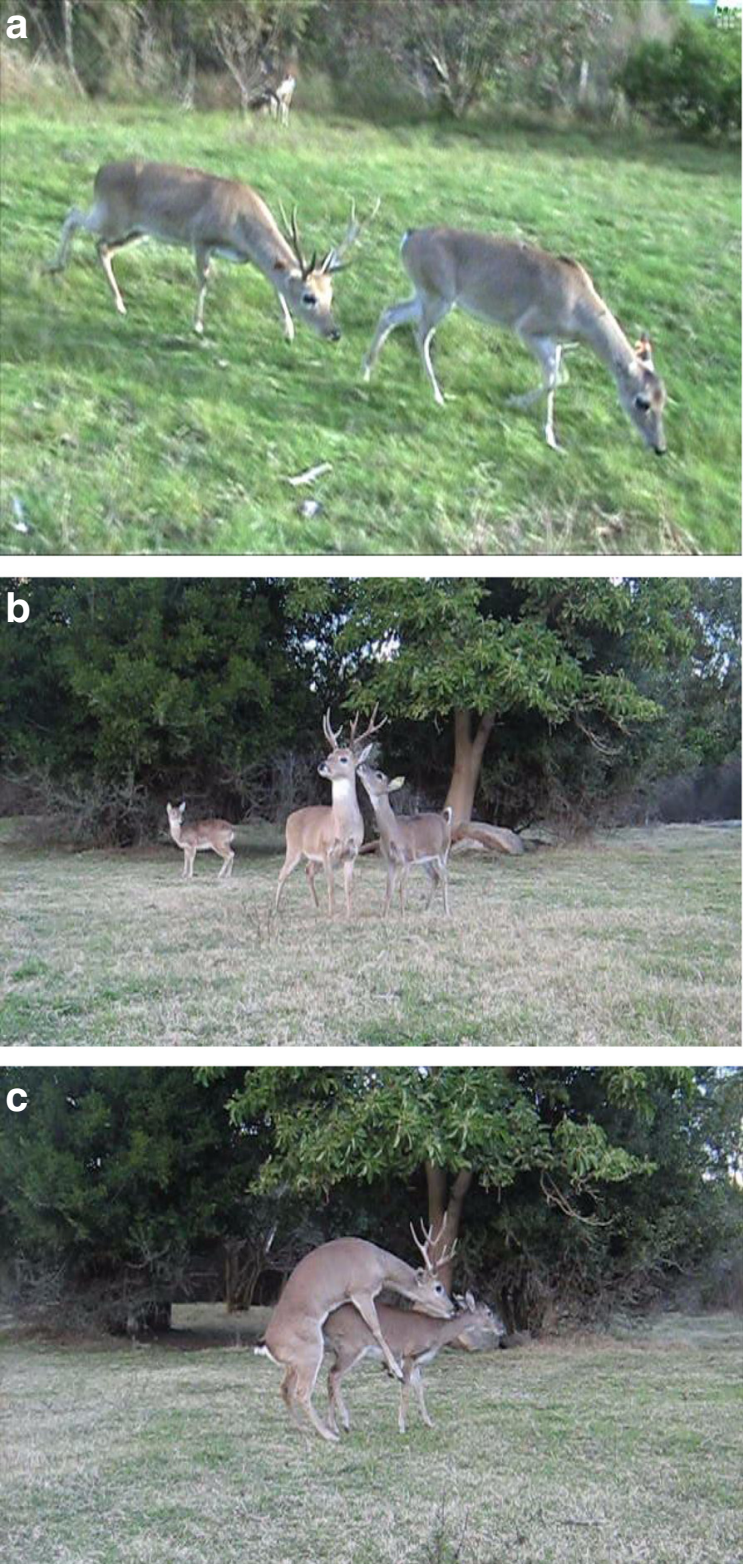
Courtship and mating behaviors of the pampas deer: a) Low stretch, b) Raised head and orientation toward the male, c) Mount.

•“Raise head”: the male was standing immobile, raising his head and keeping it in direction to the opposite side from where the female was. When the female approached the male, he raised his head and moved away, but if the female moved away, the male watched her, and immediately followed her (Figure 1).

•“Mount”: the male approached the female from the side or the back, placed his chin on female’s rump displaying movements of the tongue. The male mounted the female from the rear quarters, placing his slightly bent forelegs on either side of the female’s flank. He performed quick pelvic movements while licked or bite the female’s neck (Figure 1). We assumed ejaculation is a consequence of a set of intense abdominal muscle contractions of high frequency and low amplitude, sometimes ending with a deep thrust.

•“Dismount”: after mating, the male got down and stayed immobile, or sniffed the female’s anogenital area. He kept his tail up and directed dorsally, showing the white side of the tail.

The female sexual ethogram included:

•“Scamper”: the female ran next to the male, sometimes circling him, with her ears back and her tail up, maintaining the neck down and stretched. She approached and moved away from the male repeatedly.

•“Orientation toward the male”: the female approached the male, and either sniffed or licked his body; then she slowly moved away (Figure 1). Simultaneously the female could push her head into the flanks and abdomen of the male's body.

•“Vulva exhibition”: the female remained near the male with her ears back and her tail up and directed dorsally. The tail could move or remain still. When the male licked or sniffed her anogenital area, she responded raising the tail even more. If the male did not approach her, she turned her head and looked back at him.

•“Vulvar movements”: repeated dilatations and contractions of the vulvar lips, exposing the clitoris in the direction of the male.

•“Stillness”: The female stopped and remained standing then the male chased her while she kept her tail down.

•“Move away”: the female moved away more than 1 m from the male, walking and standing, with or without exposing the vulva. She could also run away causing a male chase.

•“Lick the male penis”: while the male remained standing and exposing his penis, the female approached him and briefly licked his genitals for a few sec.

The most frequently observed male behaviors were chinning, raise head and anogenital sniffing and licking, and the most frequent female behaviors were vulval exhibition and move away (Table 2). The least frequent observed behaviors for the male and female were the flehmen, and the vulvar movement respectively.

**Table 2:** Mating periods during which each male’ and female’ behavior was observed (Total = 15)

**Behavior**	**Number of records in which was behavior observed (%)**
Males	
Mount	15 (100)
Raised head	11 (73.3)
Chinning	11 (73.3)
Anogenital sniffing and licking	11 (73.3)
Mount Attempted	10 (66.7)
Chase	10 (66.7)
Low stretch	8 (53.3)
Follow sniffing	8 (53.3)
Sniffing and licking of the body	7 (46.7)
Chivying	6 (40.0)
Ostentation	6 (40.0)
Flehmen	4 (26.7)
Females	
Vulval exhibition	11 (73.3)
Move away	11 (73.3)
Stillness	7 (46.7)
Scamper	7 (46.7)
Orientation toward the male	4 (26.7)
Vulval movement	2 (13.3)
Lick the penis of the male	2 (13.3)

Chinning was sometimes observed before mating. After mating, the female could expose the vulva, approach toward the male and scamper, remain apathetic, or licking the male’s penis. We heard vocalizations during mating only once, but it was impossible to determine if they were produced by the male or the female.

Incomplete mounts were observed in 10 (66.7 %) of the recorded periods (1.6 ± 0.5 incomplete mounts; range = 0-7) (Table 2), corresponding to mount attempts by the side, without penis penetration. After the receptive period, the male remained near the female for several hours (pos-copulative guards).

## Discussion

This is the first complete description of pampas deer sexual behavior, including courtship and mating behaviors. The limited number of observations recorded even with a long period of observations is consistent with the exceptionally short receptive period, but it may also be possible that many females came into estrus during night hours, as in other ungulates [6,22,23]. It was especially surprising to identify the extremely short estrus period, as female receptiveness ended after less than 10 min, accepting only 1 to 3 mounts with penetration. Although no data were included, González-Sierra [9] suggested that pampas deer has a very short receptive period. It is interesting that in most cases males attempted to mate the female before the recorded matings, and that during these mount attempts females stayed immobile suggesting they were receptive before mating. Considering females seem to accept matings earlier, but ended receptivity quickly after penis penetration, it seems interesting to speculate that a quick ending of estrus may be triggered by penetration during mating. Similarly, Asher *et al.*[1] reported that 30-40 % of the red deer hinds end their estrus after copulation. In other cervids such as moose and reindeer it has also been reported that few copulations (1 to 3) occur during estrus [24,25]. In several ruminant species, including some spontaneous ovulators as sheep [26], goats [27] and cows [28,29], mating reduces estrus length. Although those species are not induced ovulators, it has been proposed the existence of a neuroendocrine reflex, originated on mechanical receptors located in the female reproductive tract, that provoke an advancement of the preovulatory luteinizing hormone surge and subsequent ovulation after copulation [30]. Therefore, we could speculate that a similar mechanism may determine the end of estrus in pampas deer. Such a mechanism would be advantageous for the male as it decreases the risk for additional matings with other males thus increasing the probability of paternity and reproductive success. This may be especially important in species such as pampas deer, in which several males may cohabit in the same groups with females [17,18].

Quick copulations as those observed in pampas deer, are typical of ruminants, including other deer species [1,4,31]. This is an advantageous mechanism that probably evolved to reduce the exposure to predators. It also decreases fighting risks when competition and aggressiveness between males to access to females is intensive [32], a situation that occurs in many cervid species during the reproductive period. In this context, male guards, as those observed in pampas deer have great importance, as the presence of other males may be avoided until the end of females’ estrus [32].

Courtship and mating behaviors were similar to those previously described in other deer species [1,4,24,31,33]. A previous description of courtship in the pampas deer was reported in a thesis performed in wild animals [16], describing seven behaviors: low stretch, ostentation, chivying, chivying and nodding, anogenital sniffing, smelling urine and flehmen. Some of these behaviors were previously described, adding now the guard behavior [12]. We confirm and expand the information of previous reports on courtship behaviors with detailed descriptions, and the report of chases and follow sniffing. Different to Verdier [16], we did not observe chivying and nodding.

Considering that in this trial recordings were performed during a long time, it can be assumed that all possible courtship behaviors displayed by this species have now been recorded. As sexual behaviors are relative “fixed” characters [34] this ethogram can be considered as representative of the pampas deer courtship behavioral repertoire. However, at the same time we should be cautious considering that no male competition was allowed in this study.

Chase and guard were the behaviors more frequently observed. Grau [35] (cited by [16]) speculated that chase may be necessary to synchronize courtship with mating. Although similar guards have been reported in other cervid species [1,2,31], they were not described by Verdier [16] probably because of the difficulties of identifying animals, and thus the knowledge that the same male had stayed for long times in several periods near the same female. Probably it was simply assumed that the male was lying or standing grazing without considering the situation as part of the courtship behavior. We already explained that guards probably decrease the risk of matings from other males, and maintain the male close to the female, available for her at the moment receptiveness starts.

Estrous females were very active toward the males, displaying specific proceptive behaviors as scamper and orientation. Females from other deer species have also been described as behaviorally proceptive (the female licking the male’s body or rubbing her chin on male’s flank) [36], although other specific behaviors may differ from those observed in pampas deer (female-male mountings) [1,2,24]. Since observed only twice vulvar movements after mating seem to be extremely rare. However, it is impossible to know whether it is a low frequency behavior, or distance and position of the observers could have limited the recordings of an uneasily observable behavior. Perhaps, this explains why this behavior is not documented in other cervids.

In 67 % of the recordings in which we observed mate attempts, females stayed immobile while males failed to mate them. Therefore, we can speculate that these attempts may be part of the normal pampas deer male courtship, and probably constitute a premating stimulus. This explanation seems more appropriate than that proposed by Ungerfeld *et al*. [12], who suggested that mount attempts may be a consequence of the inexperience of the only male they observed mating.

## Conclusion

This is the first detailed report on pampas deer mating behavior. Estrus lasted for a very short period, accepting only 3 short copulations per estrus. However, female behavior during courtship can be characterized as highly proceptive.

## Competing interests

The authors declare that they have no competing interests.

## Authors’ contributions

RU defined the strategy of the trial, and JTMP have performed this work as a part of her thesis for graduating as a Veterinarian. Both authors participated in the analysis of the information, and the outcomes of the Discussion and Conclusions. JTMP wrote the initial drafts and both authors have reviewed it until the final version, which was approved by both parties.
